# Albumin-to-neutrophil-lymphocyte ratio: a novel dual-pathway biomarker for diabetic retinopathy risk assessment

**DOI:** 10.3389/fendo.2025.1687507

**Published:** 2025-11-14

**Authors:** Chongyang She, Yong Tao, Shuo Yang

**Affiliations:** Department of Ophthalmology, Beijing Chao-Yang Hospital, Capital Medical University, Beijing, China

**Keywords:** ANLR, dual-pathway biomarker, diabetic retinopathy, NHANES, risk assessment

## Abstract

**Background:**

Diabetic retinopathy (DR) represents a leading cause of blindness globally, with conventional risk factors inadequately explaining disease occurrence and progression. The albumin-to-neutrophil-lymphocyte ratio (ANLR), a composite biomarker integrating nutritional and inflammatory status, has shown predictive value in various diabetic complications but its relationship with DR remains unexplored.

**Methods:**

This cross-sectional study utilized National Health and Nutrition Examination Survey (NHANES) data from 1998–2020 cycles to investigate the association between ANLR and DR in diabetic patients with external validation in an independent study. ANLR was calculated as serum albumin divided by neutrophil-to-lymphocyte ratio. DR status was assessed through self-reporting in NHANES and ophthalmologic examination in the validation study. Survey-weighted generalized linear models were employed to evaluate associations, with participants stratified into quartiles based on ANLR values. Restricted cubic spline analysis examined nonlinear relationships, and subgroup analyses explored effect modification.

**Results:**

A total of 6,279 diabetic participants were included in the NHANES analysis and 212 in the external validation study. After multivariable adjustment, higher ANLR quartiles demonstrated significantly reduced DR risk compared to Q1: Q2 (OR = 0.59, 95%CI: 0.38-0.91, *P* = 0.018), Q3 (OR = 0.73, 95%CI: 0.47-1.13, *P* = 0.164, non-significant), and Q4 (OR = 0.57, 95%CI: 0.36-0.90, *P* = 0.017). A significant dose-response relationship was observed (*P* for trend=0.044). Restricted cubic spline analysis revealed a nonlinear L-shaped association (*P* for nonlinearity=0.033), with rapid risk reduction at ANLR values <20 and plateau thereafter. The external validation study confirmed these findings with stronger associations (Q4 vs Q1: OR = 0.31, *P* = 0.010; *P* for trend=0.005) and demonstrated progressively lower ANLR levels across DR severity stages (no DR, NPDR, PDR; *P* for trend=0.017). Subgroup analyses identified significant interactions for sex, BMI, apolipoprotein B, and HDL-C, with stronger protective effects observed in females, individuals with higher BMI, and those with favorable lipid profiles.

**Conclusion:**

ANLR demonstrates a significant inverse association with DR risk and severity in diabetic patients, exhibiting a nonlinear dose-response relationship validated across independent populations. As an easily obtainable biomarker integrating inflammatory and nutritional status, ANLR may serve as a valuable tool for DR risk stratification and early identification of high-risk patients, potentially guiding personalized prevention strategies.

## 1 Introduction

Type 2 diabetes mellitus has emerged as a global public health concern. According to the International Diabetes Federation (IDF) report, approximately 537 million adults worldwide had diabetes in 2021, with this figure projected to reach 783 million by 2045 (1). Among all diabetic complications, diabetic retinopathy (DR) represents one of the most common and severe microvascular complications, constituting a leading cause of blindness in adults (2). Studies demonstrate that approximately one-third of diabetic patients develop varying degrees of retinopathy. After 20 years of disease duration, nearly all type 1 diabetic patients and over 60% of type 2 diabetic patients develop DR (3). The progression of DR not only severely compromises patients’ visual acuity and quality of life but also imposes substantial economic burden on healthcare systems (4). Despite the identification of several risk factors for DR, such as hyperglycemia, hypertension, diabetes duration, and dyslipidemia (5), these conventional risk factors inadequately explain the occurrence and progression of DR. Furthermore, existing predictive models remain limited by insufficient sensitivity and specificity (6). Therefore, the exploration of novel predictive biomarkers is crucial for early prevention and intervention of DR.

Serum albumin is a key marker for assessing nutritional status and is closely linked to DR risk. Research shows that patients with hypoalbuminemia have higher incidence and progression rates of DR (7). More importantly, under diabetic conditions, blood-retinal barrier dysfunction leads to abnormal albumin leakage into retinal tissues, a process that is not only an important mechanism of DR pathological changes but also makes serum albumin levels a sensitive indicator reflecting retinal microvascular integrity (8). Inflammation plays an equally crucial role in DR development, and the neutrophil-to-lymphocyte ratio (NLR), as a marker reflecting systemic inflammatory status, increases significantly with the progression of DR severity. Research has found that NLR can effectively identify patients with severe DR, demonstrating good diagnostic value (9). Multiple studies have confirmed the association between elevated NLR and DR occurrence (10). However, using NLR or albumin alone to predict DR has limitations. Given the complexity of DR pathogenesis, which involves the interaction of multiple pathological processes including metabolism, inflammation, and vascular changes, single biomarkers are difficult to comprehensively reflect disease risk (10). Therefore, composite indicators integrating nutritional and inflammatory information may have better predictive value in DR risk assessment. The albumin-to-neutrophil-lymphocyte ratio (ANLR), a composite biomarker that simultaneously reflects nutritional status and inflammatory levels, has demonstrated advantages in risk prediction for diabetic microvascular complications such as diabetic foot ulcers (11), but its application to DR risk assessment has not been investigated.

To address this knowledge gap, we utilized the National Health and Nutrition Examination Survey (NHANES) database, which represents a nationwide survey program designed to assess the health and nutritional status of the US population. Its comprehensive clinical, biochemical, and nutritional data provide an ideal platform for investigating nutrition-related diseases. Our study aimed to explore the relationship between ANLR and DR in the diabetic population. Specifically, we sought to (1): evaluate the dose-response relationship between ANLR and DR occurrence risk (2); explore the heterogeneity of this association across different subgroup populations (3); validate the ANLR-DR association and examine its relationship with DR severity in an independent study. Our findings will contribute to a better understanding of the interplay between nutrition and inflammation in DR pathogenesis, provide novel insights for clinical risk stratification of diabetic patients, and offer scientific evidence for DR prevention and intervention strategies.

## 2 Methods

### 2.1 Study design

This study employed a cross-sectional design using data from the NHANES. NHANES is a nationally representative, multistage probability sampling survey conducted by the National Center for Health Statistics (NCHS) to assess the health and nutritional status of the civilian, non-institutionalized US population. We included data from the 1998–2020 cycles. All NHANES protocols were approved by the NCHS Research Ethics Review Board, and written informed consent was obtained from all participants. For external validation, we utilized a cross-sectional dataset from Beijing Chaoyang Hospital, Capital Medical University. Diabetic patients were consecutively enrolled from the Department of Ophthalmology between January 2023 and December 2023, comprising diabetic patients with detailed retinal examination and laboratory data. Diabetes diagnosis followed the same American Diabetes Association criteria as the NHANES cohort (12). The external validation study was approved by the hospital’s ethics committee, and all participants provided informed consent.

### 2.2 Study population

Participants were identified as having diabetes based on any of the following criteria (1): fasting glucose ≥7.0 mmol/L (2); random glucose ≥11.1 mmol/L (3); glycated hemoglobin (HbA1c) ≥6.5% (4); current use of antidiabetic medications; or (5) self-reported physician diagnosis of diabetes. These diagnostic criteria are consistent with the American Diabetes Association clinical guidelines (13).

**Figure 1:**
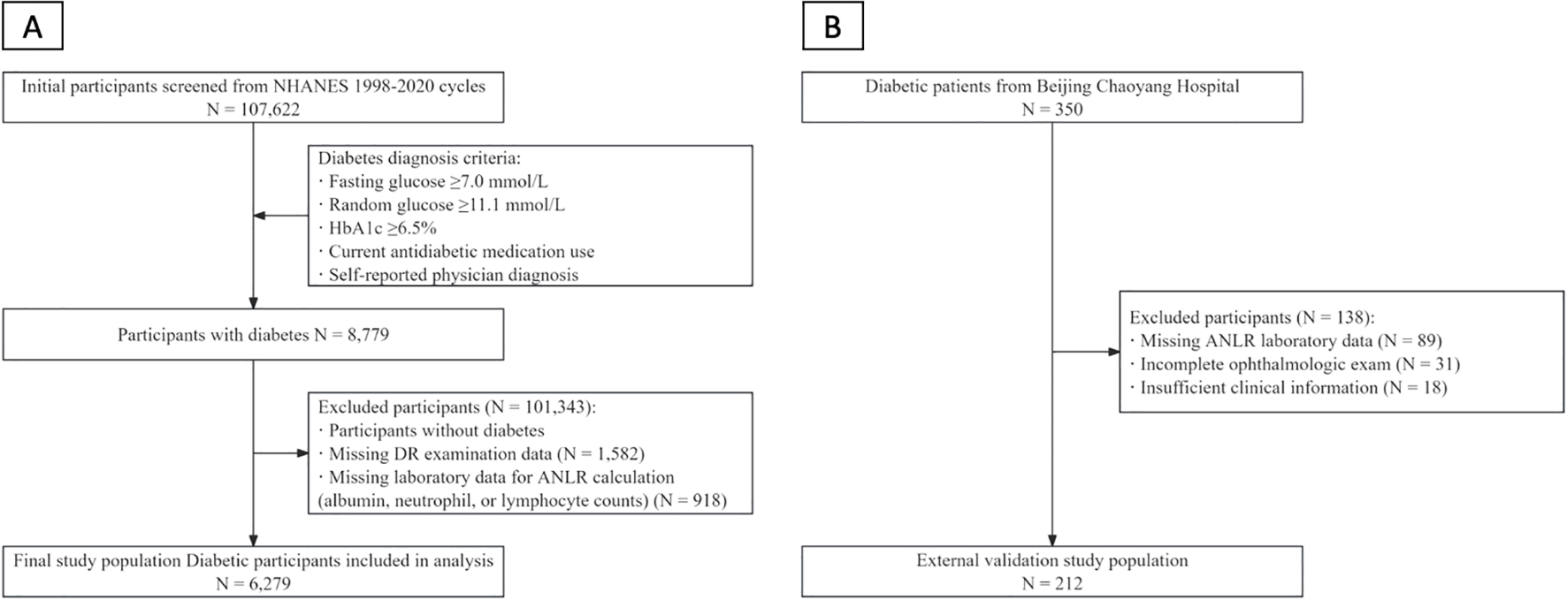
Patient selection flowchart. **(A)** NHANES study population selection. **(B)** External validation study population selection from Beijing Chaoyang Hospital.

In the NHANES study, a total of 107,622 participants were initially screened from the NHANES 1998–2020 cycles. Exclusion criteria included participants without diabetes (n=98,843), missing data on DR examination (n=1,582), and missing laboratory data for calculating ANLR, including albumin, neutrophil, or lymphocyte counts (n=918). After applying the inclusion and exclusion criteria, 6,279 diabetic participants were included in the final analysis (**Figure 1A**).

For the external validation study, a total of 350 diabetic patients were initially screened from Beijing Chaoyang Hospital. Exclusion criteria included missing laboratory data for ANLR calculation (n=89), incomplete ophthalmologic examination, and insufficient clinical information (n=18). After applying the inclusion and exclusion criteria, 212 diabetic participants were included in the external validation analysis (**Figure 1B**).

### 2.3 Variable definitions

In this study, we utilized the ANLR as the primary exposure variable. ANLR was calculated as serum albumin divided by the NLR, where NLR represents the ratio of neutrophil count to lymphocyte count. This indicator simultaneously reflects an individual’s nutritional status and inflammatory level, providing a more comprehensive assessment of patients’ metabolic and immune status compared to using albumin or NLR alone.

DR status served as the primary outcome variable in this study. In the NHANES survey, DR was assessed through participants’ responses to the questionnaire item “Has diabetes affected your eyes or have you ever had retinopathy?” Participants answering “yes” were defined as having DR. This self-report assessment method has been widely adopted in multiple NHANES-based studies of diabetic complications, offering convenience and reasonable reliability. In the external validation study, DR diagnosis and severity grading were determined through comprehensive ophthalmologic examination including fundus photography and optical coherence tomography, with cases classified as no DR, non-proliferative diabetic retinopathy (NPDR), or proliferative diabetic retinopathy (PDR) according to the International Clinical Diabetic Retinopathy Disease Severity Scale. Both ANLR calculation and DR assessment were performed during the same study visit in both cohorts.

### 2.4 Statistical analysis

For the NHANES dataset, statistical analyses accounted for the complex sampling design of NHANES, incorporating sampling weights, stratification, and clustering to ensure national representativeness of the data. According to NHANES analytical guidelines, sampling weight adjustments were applied for combined survey cycles.

Study participants were divided into quartile groups (Q1-Q4) based on ANLR values. In the NHANES study, continuous variables were presented as weighted means (95% confidence intervals), and categorical variables as weighted percentages (95% confidence intervals). All analyses incorporated survey weights to account for the complex sampling design. For the external validation study, continuous variables were presented as means ± standard deviations, and categorical variables as frequencies and percentages.

Survey-weighted generalized linear models (SWGLMs) were employed to evaluate the association between ANLR and DR in the NHANES dataset, with ANLR analyzed both as a continuous variable and by quartile groupings, calculating P-values for trend. For the external validation study, conventional logistic regression models were used. We constructed three models: Model 1 was unadjusted; Model 2 adjusted for age, sex, and race; Model 3 further adjusted for body mass index (BMI), smoking status, alcohol consumption, systolic blood pressure (SBP), triglycerides (TG), total cholesterol (TC), HbA1c, diabetes duration, and cardiovascular disease (CVD) (including congestive heart failure, coronary heart disease, angina, heart attack, and stroke). Covariates in Model 3 were selected based on clinical significance and variance inflation factor (VIF) analysis to avoid multicollinearity (VIF < 10).

To assess potential nonlinear relationships between ANLR and DR, we employed restricted cubic spline (RCS) analysis, treating ANLR as a continuous variable after adjusting for all covariates in Model 3. In the external validation study, we examined ANLR levels across different DR severity groups (no DR, NPDR, PDR) using analysis of variance (ANOVA) with trend analysis to assess the dose-response relationship between ANLR and DR severity progression.

To explore heterogeneity in the ANLR-DR relationship across different subgroups, we conducted subgroup analyses stratified by sex, race (non-Hispanic White, Hispanic, other), age (<60 years, ≥60 years), BMI (<23.9, ≥23.9), smoking status, SBP (≤140mmHg, >140mmHg), diastolic blood pressure (DBP) (≤90mmHg, >90mmHg), and glomerular filtration rate (GFR) (<60mL/min/1.73m², ≥60mL/min/1.73m²). Within each subgroup, SWGLMs adjusted for all covariates in Model 3 were fitted, excluding the stratifying variable itself. Interaction testing was performed by including interaction terms between ANLR and stratifying variables in the models, with interaction P-values <0.05 considered indicative of significant subgroup differences.

All statistical analyses were conducted using R software version 4.3.2 and EmpowerStats software version 10.0. Two-sided *P* < 0.05 was considered statistically significant.

## 3 Results

### 3.1 Baseline characteristics

**Table 1** presents the baseline characteristic differences among ANLR quartiles in the NHANES study. The study revealed significant age differences among groups (*P* < 0.001), with mean ages of 62.1 years in Q1 and 56.8 years in Q4. Sex distribution showed statistically significant differences among groups (*P* < 0.001), with female proportions of 42.9% in Q1 and 55.6% in Q4. race distribution also demonstrated significant differences (*P* < 0.001), with non-Hispanic Whites comprising 71.8% in Q1 and 48.5% in Q4. Regarding physiological and laboratory parameters, BMI (*P* < 0.001), white blood cell count (*P* < 0.001), and urinary albumin creatinine ratio (UACR) (*P* < 0.001) were highest in Q1 and lowest in Q4. Conversely, DBP (*P* < 0.001), red blood cell (RBC) count (*P* = 0.004), hemoglobin (*P* = 0.001), total protein (*P* < 0.001), TC (*P* < 0.001), low-density lipoprotein cholesterol (LDL-C) (*P* < 0.001), high-density lipoprotein cholesterol (HDL-C) (*P* = 0.001), and GFR (*P* < 0.001) were lowest in Q1 and highest in Q4. SBP, platelet count (PLT), total bilirubin, tg (*P* = 0.014), vitamin D, vitamin B12, and alcohol consumption showed no statistically significant differences among groups. CVD prevalence showed significant differences among groups (*P* < 0.001), with 35.1% in Q1 and 19.5% in Q4. Smoking status also demonstrated statistical differences among groups (*P* = 0.015). Most importantly, DR incidence differed significantly among groups (*P* = 0.012), with 23.6% in Q1 and 17.7%-19.9% in the other three groups.

**Table 1:** Baseline characteristics of NHANES participants by ANLR quartiles.

Variables	ANLR				*P*-value
	Q1	Q2	Q3	Q4	
Age (years)	62.1 (61.1,63.0)	59.8 (58.9,60.8)	58.7 (57.7,59.7)	56.8 (55.8,57.8)	<0.001
BMI (kg/m²)	33.1 (32.5,33.7)	33.8 (33.1,34.4)	32.6 (32.1,33.1)	31.2 (30.7,31.7)	<0.001
SBP (mmHg)	130.1 (128.6,131.6)	131.5 (129.9,133.1)	129.3 (127.8,130.9)	130.1 (128.5,131.7)	0.224
DBP (mmHg)	67.0 (66.1, 67.9)	69.6 (68.6, 70.5)	69.7 (68.7, 70.8)	71.1 (70.3, 72.0)	<0.001
WBC (10^9^/L)	8.5 (8.3, 8.6)	7.9 (7.7, 8.0)	7.5 (7.3, 7.6)	7.1 (6.9, 7.3)	<0.001
RBC (10^9^/L)	4.6 (4.6, 4.6)	4.7 (4.6, 4.7)	4.7 (4.6, 4.7)	4.7 (4.6, 4.7)	0.004
Hemoglobin (g/dL)	13.8 (13.6, 13.9)	14.0 (13.9, 14.1)	14.1 (14.0, 14.2)	14.0 (13.9, 14.1)	0.001
PLT (10³/μL)	240.1 (234.5, 245.7)	246.6 (240.9, 252.2)	246.5 (241.4, 251.6)	249.9 (245.0, 254.9)	0.096
Total Bilirubin (mg/dL)	10.7 (10.4, 11.1)	10.5 (10.1, 10.9)	10.4 (10.0, 10.8)	10.4 (10.0, 10.7)	0.606
Total Protein (g/dL)	70.1 (69.8, 70.5)	70.8 (70.4, 71.1)	71.4 (71.0, 71.8)	72.6 (72.2, 73.0)	<0.001
TC (mmol/L)	4.5 (4.4, 4.6)	4.6 (4.6, 4.7)	4.8 (4.7, 4.9)	5.0 (4.9, 5.1)	<0.001
TG (mmol/L)	2.0 (1.9, 2.1)	2.2 (2.1, 2.3)	2.4 (2.2, 2.5)	2.2 (2.1, 2.4)	0.014
LDL(mmol/L)	2.5 (2.4, 2.6)	2.5 (2.4, 2.6)	2.7 (2.6, 2.8)	2.8 (2.7, 2.9)	<0.001
HDL(mmol/L)	1.2 (1.2, 1.2)	1.2 (1.2, 1.2)	1.2 (1.2, 1.3)	1.3 (1.3, 1.3)	0.001
GFR (mL/min/1.73m²)	73.9 (71.9, 75.9)	81.1 (79.4, 82.8)	84.6 (83.0, 86.1)	86.6 (84.9, 88.3)	<0.001
UACR (mg/g)	251.9 (194.4, 309.3)	126.9 (97.7, 156.1)	128.2 (82.6, 173.8)	63.1 (41.4, 84.8)	<0.001
Vitamin D (ng/mL)	69.2 (66.2, 72.2)	68.3 (65.6, 71.1)	66.1 (63.0, 69.2)	67.2 (64.5, 69.9)	0.378
Vitamin B12 (pg/mL)	509.4 (422.1, 596.7)	459.7 (365.2, 554.3)	460.8 (418.5, 503.0)	446.1 (420.7, 471.4)	0.482
Sex					<0.001
Male	57.1 (54.0, 60.2)	54.0 (50.3, 57.7)	49.7 (45.9, 53.5)	44.4 (40.9, 47.9)	
Female	42.9 (39.8, 46.1)	46.0 (42.3, 49.7)	50.3 (46.5, 54.1)	55.6 (52.1, 59.1)	
Race					<0.001
Non-Hispanic white	71.8 (68.1, 75.3)	66.2 (62.7, 69.5)	58.9 (54.4, 63.2)	48.5 (44.2, 52.8)	
Hispanic	12.3 (10.0, 15.0)	13.9 (11.7, 16.5)	18.0 (15.0, 21.4)	16.5 (14.1, 19.3)	
Others	15.9 (13.5, 18.6)	19.9 (17.6, 22.5)	23.2 (20.4, 26.2)	35.0 (31.1, 39.0)	
Current smoking	55.0 (51.7, 58.2)	52.2 (48.8, 55.5)	50.7 (46.4, 55.1)	46.6 (42.9, 50.2)	0.015
Alcohol consumption	68.4 (64.1, 72.4)	63.7 (60.0, 67.3)	64.8 (60.7, 68.7)	63.7 (59.5, 67.7)	0.240
CVD	35.1 (31.9, 38.4)	28.4 (25.2, 31.9)	25.0 (21.7, 28.6)	19.5 (16.3, 23.1)	<0.001
DR	23.6 (21.0, 26.5)	17.7 (15.7, 20.0)	19.9 (17.0, 23.1)	19.6 (16.9, 22.6)	0.012

For continuous variables: survey-weighted mean (95% CI), P-value was by survey-weighted linear regression (svyglm).

For categorical variables: survey-weighted percentage (95% CI), P-value was by survey-weighted Chi-square test (svytable).

The external validation study (**Table 2**) showed similar baseline patterns, with 212 diabetic participants demonstrating comparable age distribution across ANLR quartiles and consistent trends in laboratory parameters. Notably, DR prevalence showed a descending pattern from Q1 (45.3%) to Q4 (22.6%), reinforcing the inverse relationship observed in the NHANES dataset.

**Table 2:** Baseline characteristics of external validation study participants by ANLR quartiles.

Variables	ANLR				*P*-value
	Q1	Q2	Q3	Q4	
Age (years)	63.8 ± 12.1	59.3 ± 12.0	63.2 ± 11.3	58.4 ± 10.4	0.032
BMI (kg/m²)	24.7 ± 4.7	24.8 ± 3.7	24.9 ± 3.9	24.9 ± 4.1	0.997
SBP (mmHg)	127.8 ± 14.3	125.7 ± 14.5	127.7 ± 15.0	126.6 ± 17.4	0.876
DBP (mmHg)	74.7 ± 12.8	78.9 ± 11.9	77.7 ± 10.1	77.1 ± 11.0	0.294
WBC (10^9^/L)	6.6 ± 1.6	7.1 ± 1.6	6.7 ± 1.8	6.3 ± 1.5	0.102
RBC (10^9^/L)	4.4 ± 0.9	4.6 ± 0.6	4.6 ± 0.7	4.5 ± 0.6	0.299
Hemoglobin (g/dL)	135.8 ± 20.5	134.8 ± 18.0	131.8 ± 17.3	136.2 ± 17.0	0.593
PLT (10³/μL)	221.3 ± 49.6	236.4 ± 64.6	228.1 ± 65.3	203.4 ± 55.2	0.032
Total Bilirubin (mg/dL)	16.1 ± 7.5	13.5 ± 6.1	15.4 ± 7.1	15.4 ± 10.7	0.383
Total Protein (g/dL)	69.7 ± 5.7	70.3 ± 4.7	69.8 ± 5.6	66.3 ± 5.8	<0.001
TC (mmol/L)	4.4 ± 1.1	4.6 ± 1.2	4.7 ± 1.1	4.5 ± 1.0	0.348
TG (mmol/L)	1.5 ± 0.8	1.6 ± 0.8	1.6 ± 0.8	1.5 ± 0.9	0.659
Male	28 (52.8)	29 (54.7)	25 (47.2)	24 (45.3)	0.733
Current smoking	15 (28.3)	15 (28.3)	9 (17.0)	9 (17.0)	0.275
Alcohol consumption	16 (30.2)	15 (28.3)	19 (35.9)	23 (43.4)	0.356
CVD	10 (18.9)	8 (15.1)	9 (17.0)	15 (28.3)	0.328
DR	24 (45.3)	20 (37.7)	15 (28.3)	12 (22.6)	0.066

### 3.2 Association between ANLR and DR

In weighted logistic regression analysis, we assessed the association between ANLR and DR (**Table 3**). In the NHANES study, when ANLR was treated as a continuous variable, the crude model (Model 1) revealed an inverse association with DR occurrence (OR = 0.99, 95%CI: 0.99-1.00, *P* < 0.001). This association persisted after adjustment for demographic variables including age, sex, and race (Model 2: OR = 0.99, 95%CI: 0.98-1.00, *P* < 0.001). However, in the fully adjusted model (Model 3) incorporating age, sex, race, BMI, smoking status, alcohol consumption, SBP, TG, TC, HbA1c, diabetes duration, and CVD, the association was attenuated and no longer statistically significant (OR = 0.99, 95%CI: 0.98-1.01, *P* = 0.243).

**Table 3:** Multivariable analysis of ANLR and DR risk in NHANES and external validation studies.

Variables	Model 1		Model 2		Model 3	
	OR (95%CI)	*P* value	OR (95%CI)	*P* value	OR (95%CI)	*P* value
NHANES Study						
Continuous	0.99 (0.99, 1.00)	**<0.001**	0.99 (0.98, 1.00)	**<0.001**	0.99 (0.98, 1.01)	0.243
Q1	—		—		—	
Q2	0.77 (0.65, 0.91)	**0.002**	0.76 (0.65, 0.90)	**0.001**	0.59 (0.38, 0.91)	**0.018**
Q3	0.76 (0.65, 0.90)	**0.001**	0.75 (0.63, 0.88)	**<0.001**	0.73 (0.47, 1.13)	0.164
Q4	0.72 (0.61, 0.85)	**<0.001**	0.70 (0.59, 0.83)	**<0.001**	0.57 (0.36, 0.90)	**0.017**
*P* For Trend	0.90 (0.86, 0.95)	**<0.001**	0.89 (0.85, 0.95)	**<0.001**	0.86 (0.74, 1.00)	**0.044**
External Validation Study						
Continuous	0.98 (0.95, 1.00)	**0.028**	0.97 (0.95, 1.00)	**0.022**	0.97 (0.95, 0.99)	**0.020**
Q1	—		—		—	
Q2	0.73 (0.33, 1.59)	0.431	0.70 (0.31, 1.53)	0.367	0.67 (0.30, 1.51)	0.337
Q3	0.48 (0.21, 1.06)	0.072	0.47 (0.20, 1.04)	0.066	0.41 (0.18, 0.95)	**0.039**
Q4	0.35 (0.15, 0.81)	**0.015**	0.33 (0.14, 0.76)	**0.011**	0.31 (0.12, 0.74)	**0.010**
*P* For Trend	**0.008**		**0.006**		**0.005**	

Model 1: Unadjusted analysis.

Model 2: Adjusted for demographic variables including age, sex, and race/ethnicity.

Model 3: Fully adjusted model including Model 2 covariates plus BMI, smoking status, alcohol consumption, systolic blood pressure, triglycerides, total cholesterol, HbA1c, diabetes duration, and cardiovascular disease.

OR, odds ratio; CI, confidence interval; ANLR, albumin-to-neutrophil-lymphocyte ratio; DR, diabetic retinopathy; Q, quartile. The bold values indicate statistically significant results (P < 0.05).

Quartile-based analysis demonstrated a consistent pattern of reduced DR risk across higher ANLR quartiles relative to the lowest quartile (Q1). In the NHANES study, in the crude model, the odds ratios for Q2, Q3, and Q4 were 0.77 (95%CI: 0.65-0.91, *P* = 0.002), 0.76 (95%CI: 0.65-0.90, *P=*0.001), and 0.72 (95%CI: 0.61-0.85, *P* < 0.001), respectively. In the fully adjusted model (Model 3), Q2 and Q4 maintained significant associations with reduced DR risk, with odds ratios of 0.59 (95%CI: 0.38-0.91, *P* = 0.018) and 0.57 (95%CI: 0.36-0.90, *P* = 0.017), respectively, while Q3 showed a non-significant association (OR = 0.73, 95%CI: 0.47-1.13, *P* = 0.164). Tests for linear trend were statistically significant across all models (*P* for trend: Model 1<0.001, Model 2<0.001, Model 3 = 0.044), indicating a dose-response relationship between ANLR levels and DR risk.

The external validation study corroborated these findings with even stronger associations. In the fully adjusted model, ANLR as a continuous variable showed a significant inverse association with DR (OR = 0.97, 95%CI: 0.95-0.99, *P* = 0.020). Quartile analysis revealed a more pronounced protective effect in the highest quartile (Q4: OR = 0.31, 95%CI: 0.12-0.74, *P* = 0.010) with a significant dose-response relationship (*P* for trend=0.005).

### 3.3 Non-linear relationship and severity analysis

**Figure 2:**
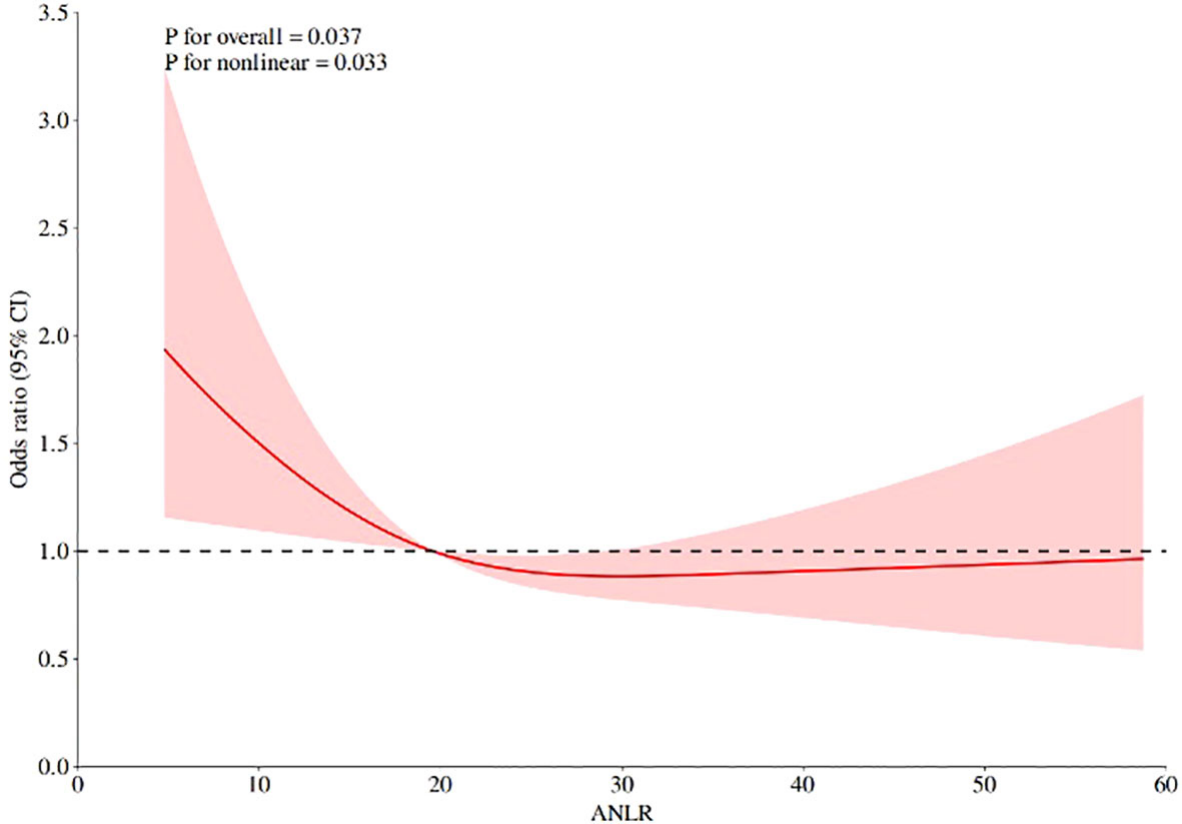
RCS analysis of the association between ANLR and DR.

To examine potential nonlinear associations between ANLR and DR, we conducted RCS analysis (**Figure 2**). The analysis revealed a significant overall association (*P* = 0.037) with evidence of nonlinearity (*P* for nonlinearity=0.033) after multivariable adjustment. The spline curve demonstrated an L-shaped pattern, characterized by a steep inverse association at lower ANLR values followed by attenuation at higher levels (**Figure 2**). The most pronounced risk reduction occurred when ANLR values were below approximately 20, beyond which the association plateaued with minimal further risk reduction. An inflection point was observed at ANLR≈20, which corresponds to specific combinations of albumin and NLR values. For instance, an albumin level of 40 g/L (within the normal range of 35–50 g/L) would correspond to an NLR of approximately 2.0 (slightly above the normal range of 1.0-3.0) at this threshold. This suggests that DR risk increases substantially when patients have either decreased albumin levels or elevated inflammatory markers that push ANLR below this threshold, where the odds ratio approached unity, suggesting a potential threshold effect. These findings indicate that the protective effect of ANLR against DR is most pronounced at lower ANLR levels, with diminishing returns at higher values.

**Figure 3:**
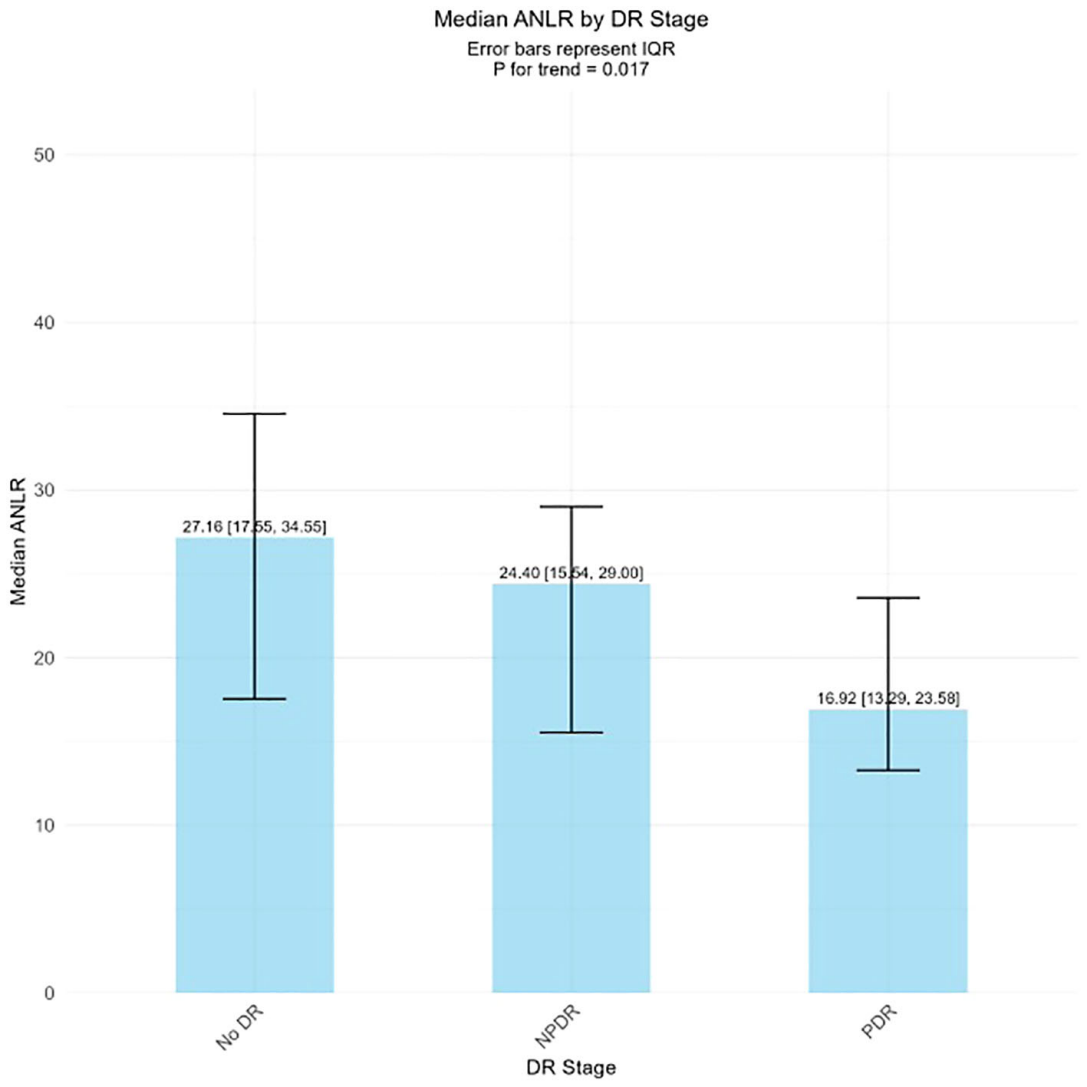
ANLR levels across different DR stages.

The external validation study further confirmed the dose-response relationship between ANLR and DR severity (**Figure 3**). ANLR levels showed a significant descending pattern across DR severity groups: No DR, NPDR, and PDR groups demonstrated progressively lower mean ANLR values, with a statistically significant trend (*P* for trend = 0.017). This finding reinforces the inverse association between ANLR and DR progression, supporting the threshold effect observed in the RCS analysis.

### 3.4 Subgroup analysis and interaction effects

Subgroup analyses were conducted to evaluate effect modification across different population strata (**Table 4**). Significant interactions were observed for sex (*P* = 0.018), BMI (*P* = 0.012), apolipoprotein B (*P* = 0.021), and HDL-C (*P* < 0.001), indicating that the association between ANLR and DR varied significantly across these subgroups. Among subgroups with significant interactions, the inverse association between ANLR and DR was significant among females (OR = 0.99, 95%CI: 0.98–0.99, *P* = 0.001), participants with BMI≥23.9 kg/m² (OR = 0.99, 95%CI: 0.98–1.00, *P* = 0.002), apolipoprotein B<1.2 g/L (OR = 0.99, 95%CI: 0.98–1.00, *P* = 0.056), and HDL-C≥1.0 mmol/L (OR = 0.98, 95%CI: 0.98–0.99, *P* < 0.001), while no significant associations were observed in the corresponding counterpart subgroups. No significant interactions were detected for race, age, smoking status, or blood pressure parameters (all *P*>0.05). However, stratified analyses revealed significant associations in certain subgroups, including participants aged ≥60 years (*P* = 0.013), and those with DBP ≤ 90 mmHg (*P* = 0.043).

**Table 4:** Subgroup analysis of the association between ANLR and DR in NHANES participants.

Variables	ANLR		
	OR 95%CI	P value	*P* for interaction
Sex			**0.018**
Male	1.00 (0.99, 1.00)	0.714	
Female	0.99 (0.98, 0.99)	0.001	
Race			0.056
Non-Hispanic white	1.00 (0.99, 1.01)	0.929	
Hispanic	1.00 (0.99, 1.01)	0.813	
Others	0.98 (0.98, 0.99)	<0.001	
Age			0.288
<60 years	1.00 (0.99, 1.01)	0.538	
≥60 years	0.99 (0.98, 1.00)	0.013	
BMI			**0.012**
<23.9kg/m²	1.01 (1.00, 1.02)	0.148	
≥23.9kg/m²	0.99 (0.98, 1.00)	0.002	
Current smoking			0.815
No	0.99 (0.98, 1.00)	0.061	
Yes	0.99 (0.99, 1.00)	0.121	
SBP			0.716
≤140mmHg	0.99 (0.99, 1.00)	0.053	
>140mmHg	1.00 (0.98, 1.01)	0.396	
DBP			0.906
≤90mmHg	0.99 (0.99, 1.00)	0.043	
>90mmHg	1.00 (0.97, 1.02)	0.715	
Apolipoprotein B			**0.021**
<1.2g/L	0.99 (0.98, 1.00)	0.056	
≥1.2g/L	1.01 (1.00, 1.03)	0.115	
HDL-C			**<0.001**
<1.0mmol/L	1.01 (1.00, 1.02)	0.007	
≥1.0mmol/L	0.98 (0.98, 0.99)	<0.001	

The bold values indicate statistically significant results (P < 0.05).

### 3.5 Predictive performance comparison of ANLR, NLR, and albumin

To evaluate the predictive performance of ANLR compared to its individual components, we conducted ROC analysis using the combined dataset from both NHANES and external validation studies. The AUC values demonstrated that ANLR provided superior discriminatory ability for DR detection compared to albumin and NLR individually (**Table 5**). ANLR achieved an AUC of 0.539 (95% CI: 0.522-0.556), while NLR showed an AUC of 0.530 (95% CI: 0.513-0.547), and albumin had an AUC of 0.450 (95% CI: 0.433-0.467). The lower AUC for albumin reflects its inverse relationship with DR risk, consistent with the protective role of adequate nutritional status. DeLong tests revealed statistically significant differences between all pairwise comparisons (all *P* < 0.001), confirming that ANLR provides significantly better predictive performance than either component alone.

**Table 5:** ROC analysis of ANLR and its components for diabetic retinopathy prediction.

Biomarker	AUC (95% CI)	*P*-value vs Albumin	*P*-value vs NLR
Albumin	0.450 (0.433-0.467)	–	<0.001
NLR	0.530 (0.513-0.547)	<0.001	–
ANLR	0.539 (0.522-0.556)	<0.001	<0.001

## 4 Discussion

This study represents the first investigation applying ANLR to DR risk assessment, analyzing 6,279 diabetic patients from NHANES, with validation in an independent external study of 212 patients. SWGLM analysis demonstrated an inverse association between ANLR and DR risk, with higher ANLR levels associated with reduced DR risk. This association remained significant after adjustment for potential confounders in quartile-based analysis, with participants in the highest quartile showing approximately 43% lower odds of DR compared to the lowest quartile in NHANES, and an even more pronounced 69% risk reduction in the external validation study. RCS analysis further revealed a significant nonlinear relationship, characterized by an L-shaped pattern with a rapid decline in DR risk with increasing ANLR at lower values, followed by a plateau after an inflection point at approximately 20. The external validation study corroborated this dose-response relationship, demonstrating progressively lower ANLR levels across DR severity stages (*P* for trend = 0.005). Subgroup analyses identified important effect modifiers of the ANLR-DR relationship, with significant interactions observed for sex, BMI, apolipoprotein B, and HDL-C, suggesting these factors may modify the effect of ANLR on DR risk. These findings underscore the importance of individual nutritional and inflammatory status in DR pathogenesis and provide novel evidence for identifying patients at high risk for DR in clinical practice. The consistency of results across two independent populations enhances the generalizability and robustness of our findings. As a readily available parameter derived from routine laboratory measurements, ANLR may serve as a valuable tool for DR risk stratification in diabetic patients.

Previous studies have primarily investigated the prognostic value of ANLR in various diseases, particularly in conditions associated with inflammatory activation and nutritional decline. Zhou et al. demonstrated a significant inverse association between ANLR and diabetic foot ulceration (11). In a cohort of 437 patients with suspected coronary artery disease undergoing coronary computed tomography angiography, Chen et al. found that the ANLR-based nomogram (ANS) served as an independent predictor of both CAD and subclinical CAD (14). Yang et al., analyzing 2,410 acute myocardial infarction patients undergoing percutaneous coronary intervention, reported that higher ANS was associated with incident atrial fibrillation (15). These studies collectively highlight the importance of ANLR as a marker of inflammatory activation and nutritional deterioration. Given that previous research has established that inflammatory activation (16) and nutritional decline (17) are both significant factors in the development and progression of DR, ANLR may have potential utility in assessing DR risk. However, direct evidence linking ANLR and DR has been lacking until now. Our study addresses this gap by demonstrating that low ANLR levels constitute an independent risk factor for increased DR risk across two independent populations. These results are consistent with previous research on the roles of inflammatory responses and nutritional status in DR pathogenesis while offering a novel integrated biomarker perspective.

The underlying biological mechanisms linking ANLR and DR can be explained through the inflammatory and nutritional components of this integrated scoring system. Low ANLR reflects both elevated NLR and decreased albumin levels, representing active systemic inflammation and nutritional deterioration—both critical factors in DR pathogenesis.

Regarding the inflammatory component, elevated NLR indicates active systemic inflammation. Specifically, increased neutrophil activity and relative lymphopenia create a pro-inflammatory milieu that activates multiple pathological cascades. Importantly, NLR reflects not only acute inflammatory responses but also chronic subclinical inflammation that may have sustained effects on DR pathogenesis (18). Unlike transient inflammatory markers, NLR captures persistent low-grade inflammation that characterizes the pathophysiology of diabetic complications over extended periods (19). This chronic inflammatory state leads to progressive retinal vascular damage through sustained activation of inflammatory cascades, making NLR particularly valuable for predicting long-term DR risk (16). The persistent elevation of NLR in diabetic patients represents ongoing immune system dysregulation that contributes to cumulative microvascular damage, which may be more clinically relevant than acute inflammatory markers for assessing diabetic complications that develop gradually over years of disease duration (20). Activated neutrophils form neutrophil extracellular traps (NETs), which generate reactive oxygen species (ROS) and directly damage vascular endothelial cells (21). This inflammatory activation upregulates pro-inflammatory cytokines including IL-1β and tumor necrosis factor-α (TNF-α), which subsequently activate the NF-κB signaling pathway in retinal vascular endothelial cells through p38 MAPK-dependent mechanisms (22). The activated NF-κB pathway increases expression of adhesion molecules (VCAM-1, ICAM-1) and chemokines (CXCL10) in retinal microvascular endothelial cells (23). This cascade ultimately leads to vascular endothelial dysfunction, increased capillary permeability, and neovascularization—all key components in DR progression (24, 25).

Concerning the nutritional component, decreased albumin levels not only reflect nutritional status but also represent the loss of albumin’s multiple protective functions, including antioxidant, anti-inflammatory, and vascular endothelial integrity maintenance properties. Hypoalbuminemia results in vascular endothelial damage and increased microvascular fragility (26, 27). In diabetic patients, the loss of albumin’s protective function can lead to retinal vascular barrier disruption, further promoting microhemorrhage, exudation, and neovascularization (28).

The progressive decline in ANLR levels across DR severity stages reflects the cumulative effects of inflammation-nutrition imbalance during disease progression. In the early stages (from no DR to NPDR), pathological changes primarily manifest as retinal capillary basement membrane thickening, pericyte loss, and microaneurysm formation. During this process, inflammatory cytokines guide leukocyte adhesion (leukostasis), damaging vascular endothelial cells and causing extravasation of albumin and other macromolecules through damaged sites (29). This chronic low-grade inflammation and early disruption of the blood-retinal barrier jointly contribute to decreased albumin levels and elevated inflammatory markers (such as NLR), thereby reducing ANLR levels. When the disease progresses to the PDR stage, the pathological mechanisms undergo a fundamental shift, characterized by retinal neovascularization and fibroproliferation. Under conditions of retinal hypoxia, vascular endothelial growth factor (VEGF) is highly induced, particularly the sustained elevation of VEGF-A, which drives abnormal proliferation of retinal neovascularization and participates in the transition from non-proliferative to proliferative stages (30, 31). PDR patients exhibit more severe systemic inflammatory states, with inflammatory markers such as C-reactive protein (CRP) and systemic inflammatory response index (SIRI) significantly higher than NPDR and non-DR patients (32), reflecting more severe nutritional depletion and chronic low-grade inflammation. Therefore, the progressive decline in ANLR not only reflects the deterioration of inflammatory and nutritional status but also reflects the progression of DR from microvascular dysfunction (NPDR) to proliferative vascular disease (PDR). The significant trend of decreasing ANLR levels with increasing DR severity (*P* for trend = 0.017) observed in our external validation study supports this mechanistic framework.

Furthermore, the dual effects of inflammation and nutritional imbalance synergistically exacerbate oxidative stress levels. Under chronic hyperglycemic conditions, diabetic patients experience severe oxidative imbalance: increased ROS production coupled with compromised antioxidant defense systems, resulting in heightened oxidative stress and subsequent pathological changes. This oxidative stress promotes retinal capillary endothelial cell apoptosis by reducing antioxidant enzyme activities (SOD and GSH) while increasing lipid peroxidation products (MDA) (33), and by activating mitochondrial apoptotic pathways such as Bax-caspase (34). Concurrently, hyperglycemia stimulates excessive synthesis of basement membrane components including type IV collagen and fibronectin, leading to vascular basement membrane thickening (35). This process ultimately results in vascular dysfunction and retinal ischemia that characterize DR (36). Therefore, low ANLR represents a comprehensive manifestation of this triple pathological mechanism: “chronic microinflammation-nutritional dysfunction-oxidative stress imbalance.” This mechanistic understanding is further supported by the progressive decline in ANLR levels observed across DR severity stages in our validation study.

Although previous studies have separately examined the relationships between inflammation or nutrition and DR, this study is the first to combine both aspects through the comprehensive indicator ANLR. Our findings are not only consistent with previous single inflammatory or nutritional marker studies but also provide novel insights for early screening and risk stratification of diabetic microvascular complications. As a readily measurable biomarker that comprehensively reflects systemic inflammation and nutritional status, ANLR facilitates understanding of the complex pathological processes underlying diabetic microvascular complications from a more holistic systemic perspective. These findings suggest that future interventions may need to simultaneously address inflammation control and nutritional support to delay DR onset and progression. Our subgroup analyses revealed that the inverse association between ANLR and DR risk was more pronounced among females, individuals with higher BMI, those with lower apolipoprotein B, and those with higher HDL-C, suggesting these factors may modify this relationship. The sex difference may involve multiple biological mechanisms. Estrogen exerts important vascular protective effects by inhibiting mitochondrial ROS production through estrogen receptors and improving endothelial-dependent dilation (37). In diabetic animal models, 17β-estradiol significantly increases endothelial nitric oxide synthase (eNOS) and cyclic guanosine monophosphate (cGMP) levels, suppresses inflammatory cytokine expression such as TNF-α and oxidative stress, and enhances albumin-mediated vascular protection (38). This suggests that in female diabetic patients, estrogen may amplify the protective effects of ANLR by enhancing albumin’s anti-inflammatory and vascular protective functions. Additionally, female diabetic patients often exhibit different lipid metabolism patterns and body fat distribution characteristics, with higher proportions of subcutaneous fat and relatively less visceral fat (39), which may influence systemic inflammation levels and metabolic homeostasis (40). The stronger association observed in the higher BMI group may relate to the dual role of adipose tissue. While obesity is typically associated with chronic low-grade inflammation, adipose tissue as an endocrine organ also secretes anti-inflammatory adipokines such as adiponectin. Adiponectin possesses multiple protective functions including suppression of TNF-α expression, inhibition of foam cell formation, antioxidant effects, and anti-apoptotic properties (41). In overweight or obese diabetic patients with good nutritional status (high ANLR), higher BMI may reflect better nutritional reserves, enabling patients to better maintain albumin levels. Adequate albumin not only serves as a nutritional marker but also synergistically regulates inflammatory responses through its antioxidant and anti-inflammatory properties, thereby demonstrating stronger compensatory capacity when facing diabetes-related metabolic stress. This finding highlights the importance of distinguishing between well-nourished obesity and malnourished obesity in assessing risk for diabetic microvascular complications. Several methodological strengths enhance the reliability of our findings. Additionally, we validated our findings in an independent external study, demonstrating consistent results across different populations and healthcare settings. First, this study utilized the NHANES database, which employs a complex multistage stratified probability sampling design ensuring excellent population representativeness, while rigorous quality control and standardized data collection procedures guarantee result reliability. Second, we employed multiple statistical approaches to validate the ANLR-DR association, including dose-response relationship analysis and extensive subgroup analyses, enhancing the robustness of our findings. The results demonstrated that the ANLR-DR association remained stable across various subgroups. Third, the external validation study provided additional insights by examining the relationship between ANLR and DR severity stages, further supporting the clinical utility of this biomarker.

## 5 Limitations

Several limitations should be acknowledged in this study. First, the cross-sectional design precludes establishment of causal relationships between ANLR and DR. Second, although we validated our findings in an independent external study, both studies employed cross-sectional designs, and generalizability to other populations requires further validation. Additionally, the use of self-reported DR assessment in NHANES rather than objective ophthalmologic examination may have introduced misclassification bias, although the consistency of findings across both diagnostic approaches in the validation cohort strengthens our conclusions. Third, residual confounding from unmeasured variables may have influenced our results. Fourth, this study did not assess the temporal relationship between ANLR and DR, limiting our ability to infer future disease trajectories. Finally, although our findings demonstrate an association between ANLR and DR in the diabetic population, prospective studies are needed to establish causality and provide additional clinical evidence for future research.

## 6 Conclusion

In conclusion, this study demonstrates a significant inverse association between ANLR and DR risk in diabetic patients, validated across two independent populations with consistent dose-response relationships. As an easily obtainable biomarker integrating inflammatory and nutritional status, ANLR may serve as a valuable tool for DR risk stratification and early identification of high-risk patients. Prospective studies are needed to establish causality and validate its clinical utility in guiding personalized prevention strategies.

## Data Availability

The raw data supporting the conclusions of this article will be made available by the authors, without undue reservation.
